# A Risk and Decision Analysis Framework to Evaluate Future PM_2.5_ Risk: A Case Study in Los Angeles-Long Beach Metro Area

**DOI:** 10.3390/ijerph18094905

**Published:** 2021-05-04

**Authors:** Bowen He, Qun Guan

**Affiliations:** 1 Department of Civil and Environmental Engineering, Vanderbilt University, Nashville, TN 37235, USA; 2 College of Civil Engineering, Hefei University of Technology, Hefei 230009, China; grz@hfut.edu.cn

**Keywords:** PM_2.5_, risk analysis, decision analysis, expected monetary value (EMV), expected utility (EU), Los Angeles-Long Beach Metro Area (LA-LBMA)

## Abstract

This study examines the L.A.-Long Beach Metro area concerning the future risk of the PM_2.5_ concentration increase. Population expansion, economic growth, and temperature increase are incorporated to estimate the probability of the magnitude of PM_2.5_ emission increase. Three possible sectors for the reduction of PM_2.5_ emissions are considered: ocean-going vessels, refineries, and electricity-generating units. The decision of how best to allocate emissions-reduction efforts among these three sectors is analyzed using a quantitative and qualitative decision-analysis framework. For quantitative analysis, Expected Monetary Value (EMV) and Expected Utility (EU) methods are used to select the optimal sector to invest in. Based on the EMV calculation, the refineries sector is 3.5 times and 6.4 times more worthy of investment compared to the electricity-generating units and the ocean-going vessels sector, respectively. For the qualitative analysis, three criteria (investment efficiency, implementation difficulty, time to become effective) are considered in the decision-making process and sensitivity analysis is conducted to inform the ocean-going vessel sector is the optimal alternative for all possible scenarios. The refineries sector is more preferred than the electricity-generating units sector when the implementation difficulty’s weight is smaller than 50%. This study provides a valuable risk and decision analysis framework for analyzing the air pollution problem associated with the future PM_2.5_ concentration increase caused by three risk factors: population growth, economic growth, and climate change.

## 1. Introduction

California is famous for its technology and entertainment industry, as well as its poor air quality. Although the improvement of air quality in Los Angeles has ended the days of public schools frequently canceling outdoor physical education, the region still suffers from some of the worst air problems in the country [1]. With the population growth, increasing use of vehicles, growing energy consumption, as well as climate change, air quality problems will still be one of the key environmental issues to all L.A. people.

Particulate matter, fine particles, or PM_2.5_, as we usually hear, are very tiny particles that float in the air at all times [2]. They are defined as fine inhalable particles with an aerodynamic diameter less than 2.5 micrometers—100 times thinner than a human hair—that people inhale every day [2]. The reason for determining PM_2.5_ as a primary air pollution concern is that this specific type of particulate matter is smaller than other air pollutants such as PM_10_, which is also popular in air quality measurement. They generally face fewer barriers when entering human organisms such as the respiratory system and cause significant damage [3]. Although there are some other air pollution particles that are smaller than PM_2.5_, such as Ultrafine particles (UFPs) which are less than 0.1 microns in aerodynamic diameter, they are extremely difficult to measure. Thus, since this study is addressed to environmental practitioners and officials for whom information concerning air pollution is valuable when the concentration of the pollutants can be monitored and measured, and results can be applied to inform real-world decisions, PM_2.5_ is the optimal choice to represent air pollution status.

The primary sources of PM_2.5_ can be ascribed to humans’ daily activities such as combustion, transportation emissions, cooking, etc. [4]. Secondary sources include chemical reactions in the environment and industrial emissions [5]. The effects of PM_2.5_ can be divided into 3 categories: (1) health effects, (2) environmental effects, and (3) climate effects. In terms of health effects, people exposed to such PM_2.5_ particles have a higher risk of suffering from lung diseases and decreasing heart function [3,6]. Moreover, children and elderly people are more likely to have increased respiratory symptoms, including irritation of the airways and difficulty breathing [7]. From an environmental perspective, PM_2.5_ is the main cause of reduced visibility in cities [8,9]. Additionally, these particles can be carried over long distances by wind and finally settle on ground or water that may further affect the water quality of lakes and streams, breaking the nutrient balance in coastal waters and river basins as well as impacting the diversity of ecosystems [10,11]. Studies also verify that PM_2.5_ is the main cause of acid rain [12]. PM_2.5_ particles are also important sources of aerosols. They are easily mixed with air to become suspended and mobile. Li et al. [13] found that aerosols have significant and complex impacts on the regional climate system. They elaborated that heavy aerosol radiative forcing can delay and suppress the initiation and development of convective regional clouds and alter the probability and intensity of rainfall [13]. Heavy aerosol loading can also have significant radiative effects by reducing surface radiation, increasing the air temperature, and lowering the boundary layer height [13]. Based on these concerns, we use the PM_2.5_ concentration level to represent the air quality in this study.

**Figure 1:**
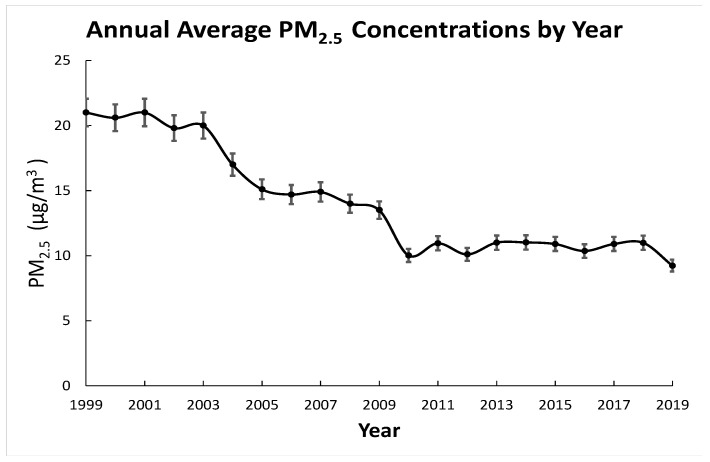
Historical annual average PM_2.5_ concentrations (µg/m^3^) in Los Angeles-Long Beach Metro Area.

With its more than 50-year-long air pollution control program, the L.A. region is undergoing a continuous improvement in air quality with a decreasing PM_2.5_ concentration. Although the PM_2.5_ concentration in the L.A.-Long Beach Metro Area (LA-LBMA) is decreasing from a historical perspective, the decreasing trend is not constant. Figure 1 reveals that there are 7 years between the years 1999–2019 that underwent a PM_2.5_ concentration rebound. A general decreasing trend can be viewed in a reduction rate of 6.9 µg/m^3^ during the 8 years from 1999 to 2007, and a smaller reduction of 3.69 µg/m^3^ from 2007 to 2015. This is an apparent indication of decreasing efficiency associated with air quality improvement strategies in the Long Beach area (Figure 1). Many studies have demonstrated that constant air quality control efforts may not be sufficient enough to maintain their efficiency in controlling the air quality [14,15], highlighting the need for dynamic actions that adapt to future uncertainties associated with population growth, economic expansion, and climate change [16,17,18,19].

Traditional methods for analyzing the PM_2.5_ level and predicting the future air pollution risk generally incorporate collecting historical data, constructing models, and applying atmospheric chemical simulations to predict the PM_2.5_ mass concentration. For example, Sun et al. [20] evaluated the PM_2.5_ related mortality at present (the 2000s) and in the future (2050s) over the continental United States by using the Environmental Benefits Mapping and Analysis Program (BenMAP-CE) and applied the Weather Research and Forecasting-Community Multiscale Air Quality (WRF-CMAQ) modeling system to simulate the atmospheric chemical fields. Zhang et al. [21] collected the historical air quality data from the China National Environmental Monitoring Center (CNEMC) and applied the AutoRegressive Integrated Moving Average (ARIMA) model to forecast the PM_2.5_ concentrations for the city of Fuzhou. Recent novel studies adopt machine learning and remote sensing to better support the PM_2.5_ concentration estimation. For instance, Li and Zhang [22] developed a hybrid remote sensing and machine learning model (RSRF) that incorporates aerosol optical depth (AOD) and the Random Forest (RF) method to predict the future PM_2.5_ concentration in the Beijing-Tianjin-Hebei region (BTH region). While these studies predicted relatively accurate numbers of future PM_2.5_ concentrations, they did not provide a generic risk analysis and decision framework to inform better decisions based on these projected numbers for local air pollution control policymakers. Farhadi et al. [23] examined the association between ambient PM_2.5_ and myocardial infarction (MI) and explored the rate of short-term exposure to PM_2.5_ and its potential effects on the risk of MI. Yu et al. [24] investigated the causal relationship between mortality and long-term exposure to a low level of PM_2.5_ concentrations using a modeling study with the difference-in-differences method. While these studies uncovered the bridge between human health risk and the PM_2.5_ concentration, they did not provide a practicable risk and decision analysis framework that incorporates the predicted simulation data to help inform reliable decisions in the uncertain future. In conclusion, we believe predicted simulation data and the knowledge of PM_2.5_ effects on human health must be analyzed within the future global context associated with industrial growth, population increase, and climate change so that the prediction is more meaningful to inform decisions [25]. In this article, we propose a risk and decision analysis framework for decision-makers to evaluate future risks associated with PM_2.5_ concentration increase. We used LA-LBMA as a case study to illustrate how the predicted numbers can be turned into real-world decisions for local decision-makers. The overarching goal of this article is to provide new insights into combining the simulation results and the risk and decision analysis framework to help local government and decision-makers make science-based decisions.

## 2. Methods

**Figure 2:**
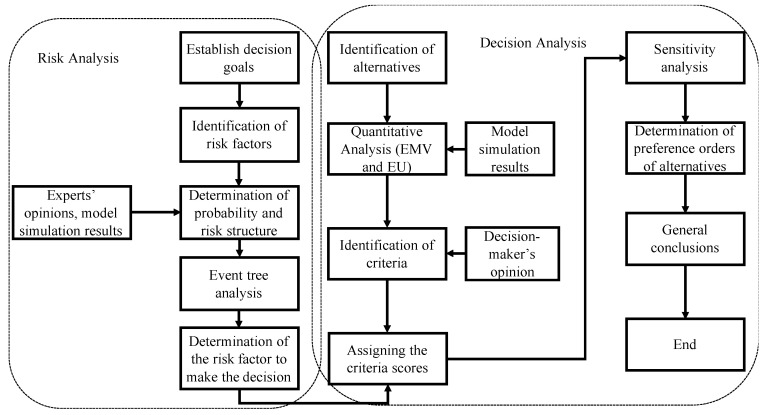
Risk and decision analysis framework associated with PM_2.5_ concentration increase.

The risk and decision analysis framework in this study includes two sections: risk analysis and decision analysis. Figure 2 displays the multi-criterion decision analysis (MCDA) process adopted in this study. In terms of risk analysis, several risk factors are first identified. In this case study, we consider three risk factors—population increase, economic growth, and climate change—that can affect the future PM_2.5_ concentration. Then, a probability and risk structure associated with each risk factor is consulted and determined based on experts’ opinions and model simulation results. Then, an event tree analysis is conducted to incorporate the information from the risk structure and experts’ opinions to determine the prior risk factor to conduct the decision analysis. A conditional probability associated with risk rating is calculated to determine the prior risk factor of concern. For the decision analysis section, we first identify several alternatives associated with the risk factor identified in the previous risk analysis process and apply both quantitative analysis and qualitative analysis to make the decision. The quantitative analysis consists of both expected monetary value (EMV) and expected utility (EU) calculations to transform the metaphysical decision’s value to the numeric measurement for comparison.

EMV is calculated by multiplying each decision’s monetary value by the corresponding probability of a PM_2.5_ concentration increase scenario and summing the total amounts for each decision based on Equation (1) [26]. The monetary value for each alternative is obtained from other relevant studies with a specific current standard.

(1) $$\mathrm{EMV}=\sum_{i=1}^{n}P_{i}*{\mathrm{MV}}_{i}$$

EMV = expected monetary value of the decision;

*n* = the total number of the scenarios associated with PM_2.5_ concentration increase;

P = the corresponding probability associated with each PM_2.5_ concentration scenario;

MV = monetary value for each alternative.

The calculation of EU applies expected utility function that incorporates decision-makers’ risk tolerance values to evaluate different alternative’s responses to decision-makers’ different risk attitudes based on the Equation (2) [26]:

(2) $$U=1-e^{-\mathrm{EMV}/R}$$

U = expected utility;

EMV = Expected Monetary Value of the decision;

R = risk tolerance.

The risk tolerance value is determined by decision-makers’ risk attitudes towards their specific decision context. The qualitative decision analysis absorbs decision-makers’ opinions on a multi-criterion decision-making process to connect the objective value of each alternative to the decision-makers’ subjective preference.

In the sensitivity analysis section, we apply a multi-criterion decision analysis (MCDA) approach to determine the preferred orders of alternatives and explore the sensitivity of each alternative to different decision-makers’ attitudes towards different criteria. A discrete sensitivity analysis is conducted based on Equation (3) [26]:

(3) $$\mathrm{DS}=\sum_{j=1}^{k}{\mathrm{CW}}_{j}{*S}_{j}$$

DS = decision score;

CW = weight assigned on each criterion;

S = score assigned on each criterion;

k = the total number of criteria considered.

A discrete sensitivity scenario analysis identifies several discrete criterion weight allocation scenarios and calculates the decision score for each of these different discrete scenarios. We conducted a continuous sensitivity analysis that computes a continuous decision score surface for each potential decision to better understand how each decision’s preference alters with the decision-maker’s attitude toward the criterion.

We use a case study in LA-LBMA to further elaborate the risk and decision analysis framework proposed here.

## 3. Case Study: PM_2.5_ Concentration Increase Risk in Los Angeles-Long Beach Metro Area (LA-LBMA)

### Study Region

**Figure 3:**
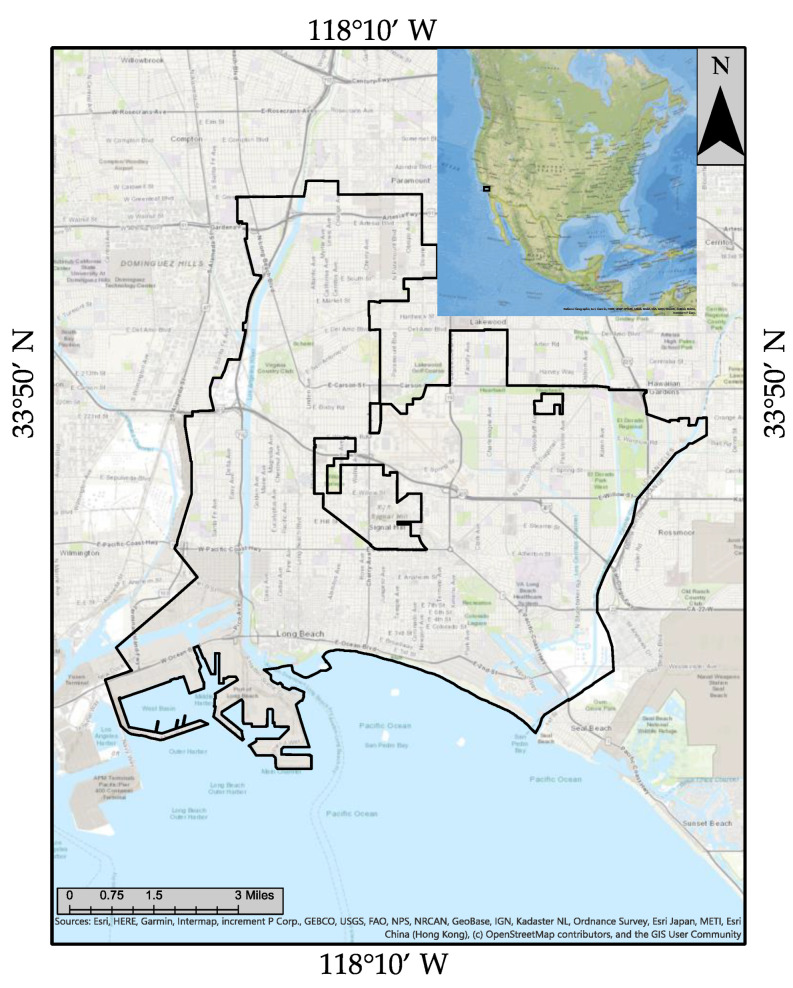
The study area depicting the Long Beach Metro area in Los Angeles County (LA-LBMA).

We chose Long Beach city in the Los Angeles metropolitan area as our case study region. It is the 43rd most-populous city in the United States, with a population of 463,218 as of 2020. Long Beach is located at 33°47′ N, 118°10′ W, about 20 miles (32 km) south of downtown Los Angeles. The city has a total area of 133,221 square kilometers, with 130,258 square kilometers of land and 2.96 square kilometers of water. Long Beach completely surrounds the city of Signal Hill. Studies reveal that ocean-going ships represent a significant source of air pollution to coastal communities such as LA-LBMA during periods with regional transport [27]. For a coastal area like LA-LBMA, the adverse influence of ship emissions on air quality can be aggravated by the onshore flow of sea breeze circulation [28]. Figure 3 depicts the city boundary of the Long Beach Metro area and the city of Signal Hill.

## 4. Risk Factors in LA-LBMA Air Quality

### 4.1. Population Increase Contributions to PM_2.5_ Concentration

**Figure 4:**
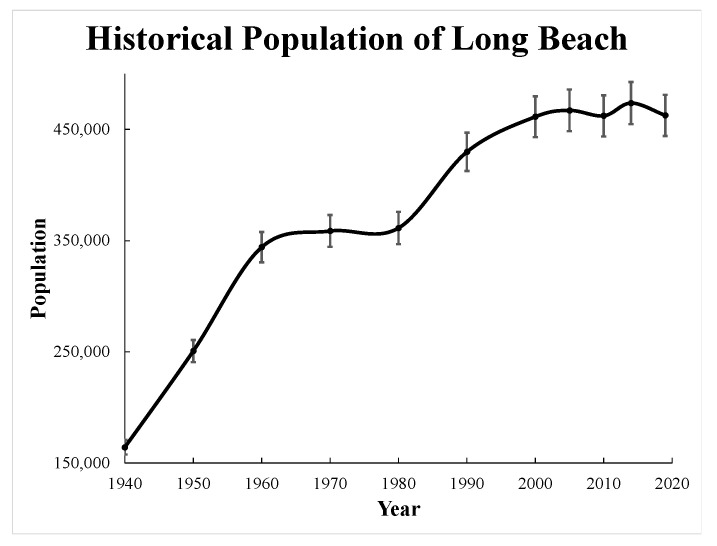
Historical population of Los Angeles-Long Beach area.

Population growth plays a very critical role in increasing the PM_2.5_ concentration level. On one hand, fine particles primarily come from vehicles, trucks, buses, and off-road vehicle exhausts that increase with population growth. Particularly, the change of vehicles and the long-range transport of air pollutants from distant sources can potentially affect air quality [29]. For instance, during long-range transport, meteorological conditions will vary and there will be sufficient time for chemical transformations to occur, allowing secondary pollutants such as ozone to form [29]. On the other hand, a larger population causes more demand for energy sourced from power plants that are operated to meet increasing needs for energy. These power plants can generate fine particles through the reaction of gases or droplets in the atmosphere. Meanwhile, PM_2.5_ is also produced by many indoor activities such as smoking, cooking, burning candles or oil lamps, and operating fireplaces and fuel-burning space heaters. Figure 4 displays that, in LA-LBMA, the population is continuing to increase, with an annual growth rate decreasing from 14.85%/year during 1890–1900 to the recent 0.61%/year during 2010–2014 [30]. If the population growth rate would be the same as in the period 2010–2014 that is 0.61%/year, the population of the Long Beach Metro area in 2026 will be 503,377.

### 4.2. Industrial Growth Contributions to PM_2.5_ Concentration

**Figure 5:**
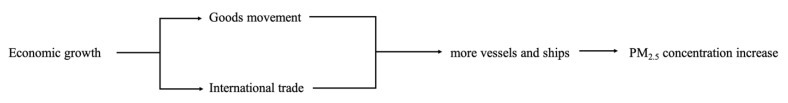
An example of economic growth indicators associated with PM_2.5_ concentration.

The Port of Long Beach, along with its twin the Port of Los Angeles, is the largest source of air pollution in the metropolitan Los Angeles area. The two ports are located in southern coastal California and have a history of over 100 years. These two ports act as major gateways for US-Asian trade, ranking as the first two busiest container ports in the world. Ships, trucks, harbor crafts, cargo-handling equipment, as well as locomotives that transport within these ports contribute many fine particles to this region. Based on Dabdub and Vutukuru’s [31] research on the air quality impacts from ship emissions, ship emissions are becoming the major sources of air pollutants near ports. Corbett and Koehler’s [32] study of emissions from ocean shipping estimated that global sulfur and nitrogen oxide emissions from international shipping are 6.49 teragram (Tg) and 6.87 Tg, respectively. Emissions of nitrogen oxides and sulfur oxides can ultimately lead to the formation of secondary pollutants such as particulate matter (PM) through a series of atmospheric chemical and physical mechanisms. Eyring et al. [33,34] concluded that the prediction of goods movement, improvement of international trade, and U.S. gross domestic product growth are important indirect indicators of ship activities, and with the high probability of increasing ship traffic in the future, ship emissions are predicted to increase significantly to further damage the air (Figure 5). Corbett et al. [35] predicted that those indicators are likely to grow by at least 6% per year; thus, ship emissions will become a significant portion of regional air pollution inventories. This study summarizes all those factors as economic uncertainties.

However, both ports have implemented many environmental programs to reduce air pollution. The air quality program includes the Clean Air Action Plan (CAAP), San Pedro Bay Standards, Technology Advancement Program, Clean Trucks Program, Green Flag Program, Green Ship Incentive Program, Shore Power, Clean Air Action Plan Awards, Annual Air Emissions Inventories, and Air Quality Monitoring. The detailed information associated with these air quality programs is summarized in Table 1.

### 4.3. Climate Change Contributes to PM_2.5_ Concentration

Recent studies have revealed that the relationship between climate change and air pollution is not a simple one-way direction—not only does air pollution cause climate change but climate change also leads to air pollution. The reason lies in the fact that climate change causes the alteration of airflow patterns. In some regions, the airflow pattern changes from circulating to static, making particulate matter accumulate and thus increasing the region’s PM_2.5_ concentration. Based on the study by California Air Resources Board (CARB), the airflow pattern in the South Coast Air Basin (SoCAB) seems to intensify the air pollution. For example, Hayes et al. [37] indicated that several airflow patterns including Calm (Type VII), Offshore (Type III), and Downslope (Type III) are dominant in summer and allow pollutants to accumulate in the region. They also verified that the formation of this airflow pattern is closely related to climate change [37]. Furthermore, numerous studies have identified the precipitation scavenging effects on aerosols and PM_2.5_ mass concentrations. Jacob and Inner [38] study revealed that PM_2.5_ concentration decreases with increasing precipitation because rainfall can sink particulate particles. Sun et al. [39] further revealed the distinct impacts of different types of precipitation on PM_2.5_ mass concentration. They found that for precipitation events with an amount of 0.1–0.5 mm, PM_2.5_ mass concentration increased with precipitation amount at a rate of 0.85 µg/m^3^ per 0.1 mm, while for precipitation events with an amount larger than 10 mm, PM_2.5_ mass concentration decreased with precipitation amount at a rate of 0.17 µg/m^3^ per 1 mm [39]. They also identified the effects of aerosol amount on the response of PM_2.5_ mass concentration to precipitation, with weak pollution prone to increase with precipitation and heavy pollution prone to decrease with precipitation [39]. Precipitation is highly dependent on regional climate patterns and temperature [40]. Chen et al. [41] summarized that wind speed, wind direction, humidity, radiation, atmospheric pressure, and planetary boundary layer height can interact with PM_2.5_ concentrations through different mechanisms, including dispersion, growth, chemical production, photolysis, and deposition of PM_2.5_. Another potential risk from climate change is wildfires induced by temperature increases. Studies reveal that wildfires generate tremendous amounts of particulate particles in the air [42].

**Figure 6:**
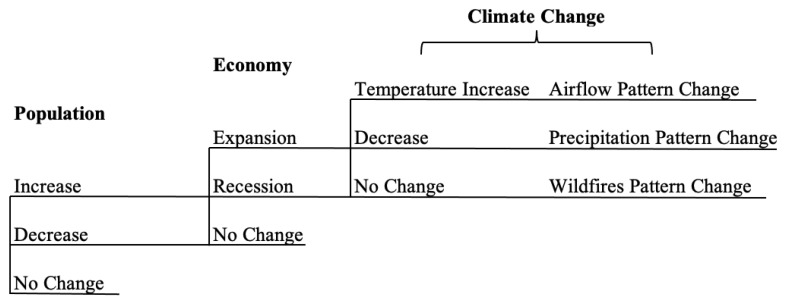
Event tree for the increase in PM_2.5_ concentration.

Given the risks described above, the probability of results associated with the increase in PM_2.5_ in LA-LBMA can be depicted in the form of an event tree, as shown in Figure 6. The event tree indicates that there are many uncertainties in the future, although we only consider three aspects in this study. Thus, risk analysis becomes vital in the current decision-making process.

## 5. Risk Analysis in LA-LBMA PM_2.5_ Increase

### 5.1. Probability and Risk Structure

Traditional mathematical models applied various approaches, including the stochastic Volatility (SV) method and the Stock–Watson (SW) method as well as the Artificial Neural Network method to forecast future PM_2.5_ concentration levels to inform decisions [43,44]. However, these models only focus on estimating the PM_2.5_ concentration and ignore the connection between the concentration and its underlying mechanisms and risk factors so that the concentration itself cannot provide a context of the future condition and has little insights for making the right decision. In this case study, we incorporate only three risk factors, population growth, economic growth, and temperature increase, for the sake of simplicity. Our goal is to elaborate the process of conducting a science-based decision-making process by analyzing the risk factors associated with future PM_2.5_ concentration increase to inform appropriate decisions based on our defined objective function.

Three risk factors that can cause future PM_2.5_ concentrations to increase are considered in this study: population, economic growth, and temperature increase. Interconnections between these three factors are ignored for simplification and we assume these three risk factors’ impacts on future PM_2.5_ concentration increase are independent.

### 5.2. Population

**Figure 7:**
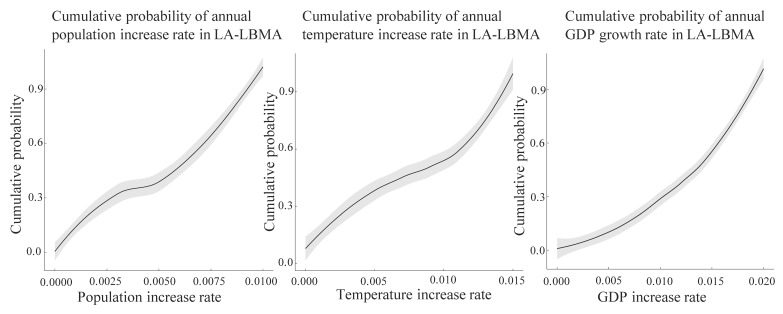
Cumulative probability of three risk factors associated with future PM_2.5_ concentration increase in LA-LBMA. (**a**) cumulative probability of annual population increase rate in LA-LBMA, (**b**) cumulative probability of annual temperature increase rate in LA-LBMA, (**c**) cumulative probability of annual GDP increase rate in LA-LBMA.

Population in the Long Beach area is expected to increase at a fairly slow rate because of the out-immigration [45]. Based on the current annual population growth rate, about 0.6%/year, the annual average projected population increase rate will be likely less than 1% in the future 10 years. The approximated cumulative probability of population increase rate in the area is based on historical data, model prediction, and experts’ assumptions [46]. Figure 7a displays our beliefs associated with the cumulative probability of annual population increase rate in LA-LBMA. The grey shaded area indicates a 95% confidence level associated with the cumulative probability distribution after consulting with the experts in the field. Detailed information regarding the expert’s consultation process is provided in the supplementary material. However, it should be noted that with the increasing knowledge of migration and population increase patterns, the cumulative probability of population increase should be dynamically updated based on the newest updated model results as well as contemporary global context.

### 5.3. Temperature

The average annual temperature in the Long Beach area is 64.8 °F [47]. Based on research by Reed [48], the temperature is increasing across Southern California, with fluctuations. Locally, the average annual temperature is predicted to rise 0.7 °F to 1 °F, around a 1.5% increase rate. The cumulative probability is based on current knowledge and predictions [47]. Figure 7b displays our beliefs associated with the cumulative probability of the annual average temperature increase rate in LA-LBMA. We also want to point out that the cumulative probability of the annual average temperature increase rate should be dynamically updated to the newest knowledge and modeling results.

### 5.4. Economy

The current GDP growth rate in LA-LBMA is around 2% and the local economic increase rate is dependent on many external factors such as international relationships and domestic political situations. In this study, we maintain a conservative attitude towards economic factors. Figure 7c displays our predictions associated with the cumulative probability of average annual GDP growth rate that is based on the current predictions and assumptions [49].

Based on these prior assumptions, we define the increased level based on the average annual increase rate and the definition is summarized in Table 2.

The current annual PM_2.5_ concentration level is around 12 µg/m^3^, approximately equal to the National Ambient Air Quality Standards (NAAQs). Based on the historical data and predictions of future PM_2.5_ concentration, we believe a very huge increase in PM_2.5_ will be unlikely. Thus, we define the increase of 20% as a high increase scenario, the increase of 10% as a medium increase scenario, and the increase of 5% as a low increase scenario. The detailed information associated with the defined level of PM_2.5_ concentration increase within 10 years is summarized in Table 3.

**Figure 8:**
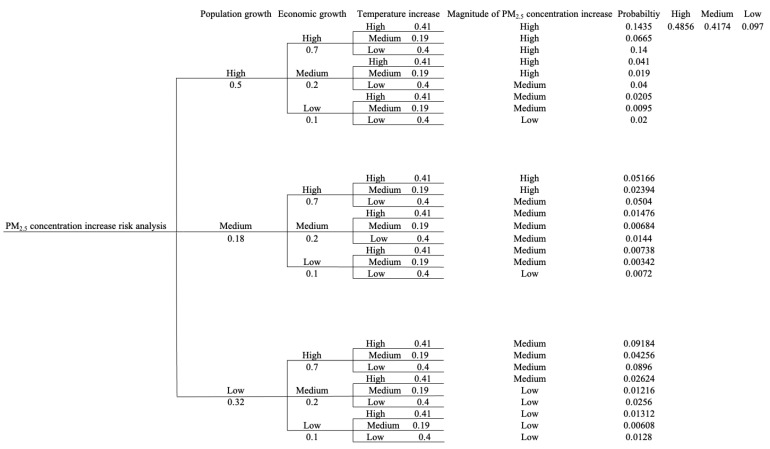
Complete event tree of PM_2.5_ concentration increase risk analysis.

Based on the information above, the complete event tree of the PM_2.5_ concentration level in the future is graphed in Figure 8. Figure 8 exhibits the potential future pathways towards PM_2.5_ concentration level increase and the probability and consequences associated with each pathway. The probability of each uncertain future scenario associated with each risk factor is calculated based on the information in Figure 7 and Table 2. For example, the high increase rate scenario for population expansion is defined as 0.6–1% based on Table 2, and Figure 7a can be consulted to derive the probability value for the high increase rate scenario for population expansion risk factor, around 50%. The final comprehensive probability of a PM_2.5_ concentration consequence that can be categorized into a high concentration scenario, medium concentration scenario, or low concentration scenario is summed by all the probabilities of the paths corresponding to that scenario. The event tree is useful for the quantitative risk assessment in this context and the prevention of a high concentration scenario can be emphasized based on the risk analysis for each path on the event tree. We also calculated two conditional probabilities to select the critical risk factor (Table 4) for our decision analysis. In this study, we define the high PM_2.5_ concentration increase as a consequence of failure that we want to avoid in the future. Based on Table 4, we select the economic growth section as our major concern in the following decision analysis framework since the conditional probability indicates that economic growth has the largest probability of causing a high PM_2.5_ concentration increase level for LA-LBMA compared to the other two risk factors.

## 6. Decision Context Definition and Decision Alternatives

Based on the risk analysis associated with the PM_2.5_ concentration increase, we have considered several high-level influential risk factors such as climate change and population, and we used the conditional probability risk rating approach to determine that the economic growth factor is the section we want to target to mitigate its effects on future PM_2.5_ concentration increase. In terms of economic growth risk factors associated with PM_2.5_ concentration, the US EPA [50] developed an approach to estimate the average avoided human health impacts, and to monetize the benefits associated with emissions of PM_2.5_ from 17 industrial sectors using the results of source apportionment photochemical modeling. The main 17 industrial sectors include: (1) aircraft, locomotives, and marine vessels; (2) area sources; (3) cement kilns; (4) coke ovens; (5) electric arc furnaces; (6) electricity-generating units; (7) ferroalloy facilities; (8) industrial point sources; (9) integrated iron and steel facilities; (10) iron and steel facilities; (11) non-road mobile sources; (12) ocean-going vessels; (13) residential wood combustion; (14) pulp and paper facilities; (15) refineries; (16) residential wood combustion; (17) taconite mines. The best and ideal scenario will be that decision-makers have unlimited resources to invest in all of these sectors and refine each of them to lower their effects on PM_2.5_ concentration risk. However, in reality, decision-makers only have limited resources and have to choose the optimal solution from all possible pathways. Thus, the decision analysis tries to answer the question: which industrial sector should be preferred to invest in among all of those sectors?

In this study, for simplification, we only analyze three sectors here: (1) ocean-going vessels; (2) electricity-generating units; (3) refineries. There are many ocean-going vessels carrying cargo going in and out of the two ports in the Long Beach area, thus making this sector more of a concern for many people. Jayaram et al. [51] illustrated that money can be spent to switch vessels to cleaner-burning fuels and operate in a lower oxide of nitrogen. For refineries, many oil refining companies are operating in the Long Beach area and money can be spent to install air cleaning and monitoring units within the factory and improve factories’ production efficiencies. Electricity-generating plants are a huge source of air pollution and money can be spent to remove sulfur from coal before combustion and improve energy conversion efficiencies.

Fann et al. [52] contributed to the interface between air pollution and human health and estimated the economic value of avoiding human health impacts caused by the ambient concentration of PM_2.5_ for each sector using the Environmental Benefits Mapping and Analysis Program (EBMAP). For each sector, a monetary value per ton of directly emitted PM_2.5_ reduced from the sector can be estimated. The information on these estimated monetary values is shown in Table 5 and all the values are based on the 2010 currency standard. It should be noted that all the monetary value per ton of directly emitted PM_2.5_ reduced from each sector should be dynamically updated to the current inflation condition and currency standard. Here, we adopt the 2010 currency standard as an example.

## 7. Decision Analysis

Based on the risk analysis conducted and three potential decision alternative sectors (ocean-going vessels, refineries, and electricity-generating units) that can be invested to mitigate the PM_2.5_ emissions, the decision analysis here serves to identify the worthiest investing sector between those alternatives. The decision analysis in this study uses two approaches: quantitative and qualitative. The quantitative approach uses monetary value and probability to generate expected monetary value (EMV). EMV is a statistical concept that calculates the average outcome of a certain decision when the future includes uncertain scenarios using the monetary concept [53]. The expected utility (EU) is calculated through the exponential utility function based on constant risk-averse tolerance. Expected utility is a weighted average of the utilities of each of its possible outcomes, where the utility of an outcome measures the extent to which that outcome is preferred, or preferable to the alternatives [54]. The qualitative approach uses potential objectives to evaluate relative weights and ranking for each alternative within a multi-criteria decision analysis setting. Sensitivity analysis is also implemented by changing the decision-maker’s risk tolerance for each of the alternatives. Finally, we obtain the relationship between the optimal decision and the decision-maker’s tolerance.

In this case, we set up the NAAQs as the standard, 12 µg/m^3^. This standard number is based on the annual arithmetic mean over 3 years set up by EPA [55]. Specifically, EPA is maintaining spatially averaged criteria to compute the annual mean, with revisions to the criteria for when the spatial averaging approach can be updated [56]. Thus, we want to point out that the approximated PM_2.5_ mass concentration calculated in this case study is in compliance with the EPA’s spatially average criteria in order to be meaningful. For the sake of clarity, we use the mass to measure the amount of the PM_2.5_ and assume that the air volume above the Long Beach area is V m^3^. The mass of PM_2.5_ needed to be reduced equals the PM_2.5_ concentration needed to be reduced times V m^3^. The potential consequence of the increase in PM_2.5_ concentration is summarized in Table 6. In Table 6, the probability associated with each scenario regarding PM_2.5_ increase projection is based on the calculation results shown in Figure 8. The value of PM_2.5_ concentration needed to be reduced for each scenario is the difference between the defined level of PM_2.5_ concentration increase for each scenario shown in Table 3 and the current NAAQs standard, 12 µg/m^3^.

### 7.1. Quantitative Decision Analysis (EMV and EU)

**Figure 9:**
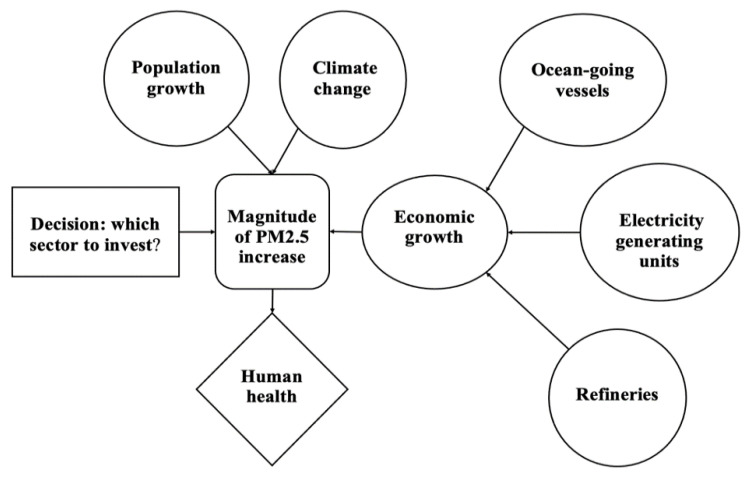
Influence diagram associated with quantitative decision analysis.

The objective of the quantitative decision analysis is to find the industrial sector that deserves the preferential investment of decision-makers. The influence diagram of this decision framework is summarized in Figure 9.

Figure 9 displays that our objective is to optimize human health that is affected by the magnitude of PM_2.5_ increase. The decision of the optimal sector to invest in affects future PM_2.5_ increases, thus further influencing human health in LA-LBMA. However, there are differences in the probability of the magnitude of PM_2.5_ increase since different sectors can influence the PM_2.5_ concentration in varying degrees. We collected several experts’ opinions on the probabilities of success for each sector and the information is summarized in Table 7. It should be noted that the probabilities of PM_2.5_ increase and success of each scenario for each alternative can also be updated with our newest knowledge and understanding.

**Figure 10:**
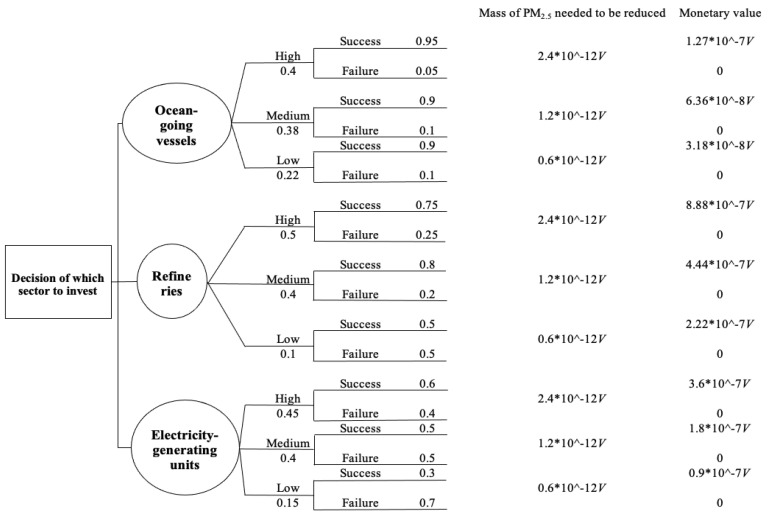
Decision tree associated with EMV analysis and calculation.

A decision tree can further exhibit the details for calculating EMV (Figure 10).

In Figure 10, the monetary value is calculated by multiplying each approximated monetary value regarding direct emission reduction of PM_2.5_ from each sector (Table 5) by the mass of PM_2.5_ that needs to be reduced (Table 6) for each PM_2.5_ increase scenario. EMV is calculated by multiplying each monetary value by the corresponding probability of a magnitude increase in PM_2.5_ concentration and summing the total amounts for each decision. The sector with the highest expected monetary value is the highest priority to invest in. Table 8 summarizes the information of the EMV calculation results.

Based on the EMV criteria, the preferred sector to invest in is refineries. However, EMV is based on a risk-neutral attitude, and optimizing the monetary value is the only criteria. If we assume the decision-maker is not risk-neutral, it is possible to obtain different outcomes based on other risk attitudes. Thus, we applied the expected utility function (Equation (2)) [26] to evaluate alternatives again to explore different alternatives’ responses to decision-makers’ different risk attitudes.

R is risk tolerance determined by the decision-maker to show the extent that the decision-maker is willing to accept risk. We set up three different common values for different risk tolerance value—R [R_1_ = 5 (risk-averse), R_2_ = 100 (risk-neutral), R_3_ = 200 (risk-tolerance)]—to elaborate this example [26]. It should be noted that the R can be adjusted based on the decision-maker’s risk attitude and the value chosen here only serves as an example. The calculation of expected utility (EU) with various risk tolerance attitudes (R) is summarized in Table 9. Based on the calculated expected utility, the refineries sector remains the most preferred alternative for all risk tolerance levels. It is interesting to notice that for R_3_ = 200, the decision-maker is indifferent between the alternative of ocean-going vessels and electricity-generating units. For all risk tolerance levels, the ocean-going vessels sector remains the least preferred sector to invest in, which contradicts with our intuition since there are two ports in the Long Beach area with tremendous vessel transportation.

### 7.2. Qualitative Assessment of Multi-Criteria Decision Analysis

The quantitative decision analysis only takes the monetary value of each sector into consideration. However, there are more criteria that should be taken into consideration when making the decision. In this study, we summarize three criteria that are closely related to this decision problem and resolve it to see whether there is a difference in choosing the optimal sector through different analysis approaches. The three criteria are summarized as below:

(1) Investment efficiency—the investment efficiency indicates the total amount of PM_2.5_ that can be reduced from the sector per one million dollars invested. More efficiency associated with investment is always preferred by the decision-maker;
(2) Implementation difficulty—the implementation difficulty indicates the difficulty of the refinement of a sector to reduce the PM_2.5_ emissions. It includes many aspects such as technical difficulties, policy obstructions, etc. Less implementation difficulty is always preferred by the decision-makers;
(3) Time to become effective—the time to become effective indicates the time needed for the sector to lower the PM_2.5_ emissions. The sector that needs less time to become effective is always preferred by the decision-makers.

Table 10 displays an example scenario associated with weight assignment of each criterion adopted in this case study.

**Figure 11:**
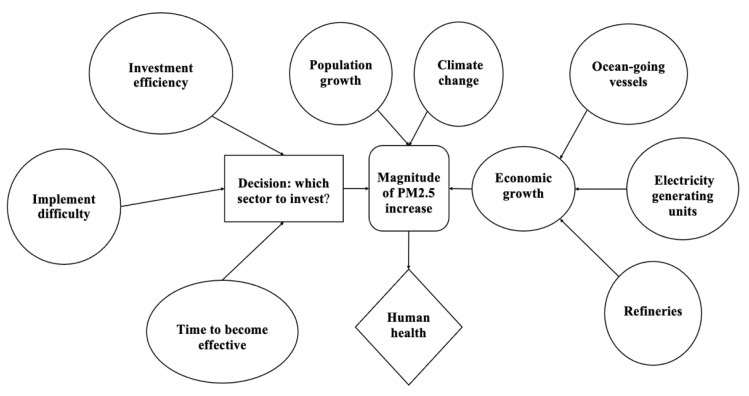
Influence diagram associated with the qualitative decision analysis.

With the additional decision criteria, the influence diagram is graphed in Figure 11.

Each criterion is assigned a range of values based on estimation and experts’ opinions. The ranges of valuation for each criterion are summarized in Table 11.

In this multi-criteria decision analysis (MCDA), the value of the three criteria corresponding to each alternative was applied and the ranking was transferred to the score value. Each ranked criterion is multiplied by its corresponding weight and summed to generate a comprehensive weighted score based on Equation (4) for each sector [26]. This alternative preference for multi-criteria decision analysis is summarized in Table 12. It should be noted that:

(4) Score=IE∗Weight(IE)+ID∗Weight(ID)+T∗Weight(T)

IE = investment efficiency;

ID = implementation difficulty;

T = time to become effective;

Weight (IE) = weight assigned on investment efficiency;

Weight (ID) = weight assigned on implementation difficulty;

Weight (T) = weight assigned on time to become effective.

Based on the qualitative analysis, the preferred sector is ocean-going vessels, which is different from the optimal sector according to the quantitative EMV and EU analysis. Nonetheless, the result above only provides a narrow scope of this decision problem since the optimal alternative depends on the way we assign the weight values of three criteria to each alternative.

### 7.3. Sensitivity Analysis

**Figure 12:**
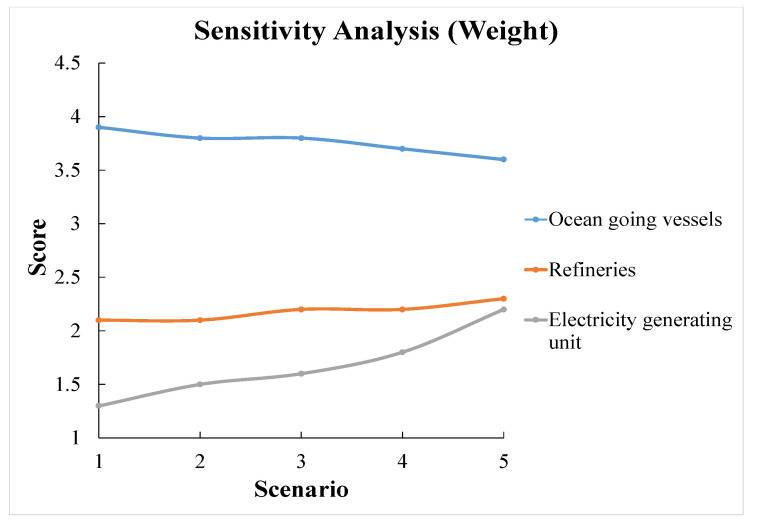
Sensitivity analysis associated with the five different scenarios of criterion weight assignment.

The sensitivity analysis is conducted to explore how the optimal decision can be influenced by the decision-makers’ different attitudes towards each criterion. First, we apply five different scenarios of weight assignment to the three criteria. The information is shown in Table 13. Figure 12 displays the sensitivity analysis results associated with the five different weight assignment scenarios. Although Figure 12 indicates that the optimal alternative always remains the ocean-going vessels, the trend displays that the preference is closely attached to decision-makers’ attitudes towards different criteria. In order to further explore the connection between decision-makers’ attitudes and alternative preferences, we apply a continuous sensitivity analysis and take all possible weight assignment scenarios into consideration. Similar to Equation (4) [26], we assume the *x*-axis is the weight of criterion investment efficiency that ranges from 0–1 and the *y*-axis is the weight of implementation difficulty that ranges from 0–1. For each alternative, the equation to calculate the continuous preference score is:

(5) Score=score(IE)∗x+score(ID)∗y+score(T)∗(1−x−y)

Score (IE) = score assigned on investment efficiency criterion;

Score (ID) = score assigned on implementation difficulty criterion;

Score (T) = score assigned on time to become effective criterion;

x = weight assigned on investment efficiency criterion;

y = weight assigned on implementation difficulty criterion.

**Figure 13:**
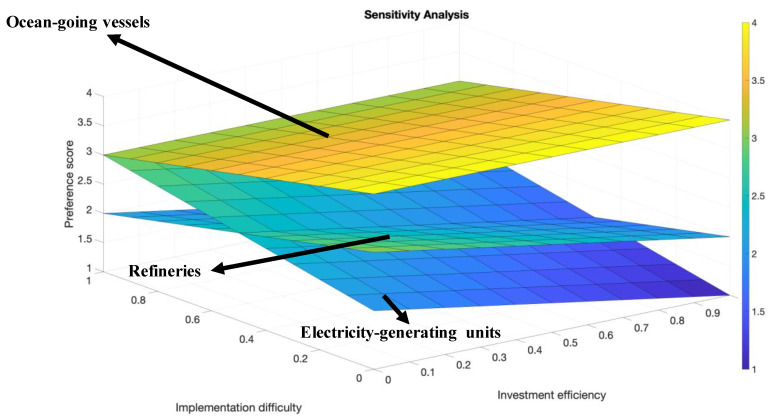
Sensitivity analysis associated with continuous criterion weight scenarios.

Figure 13 exhibits that the optimal sector will change under different weight assignment scenarios. The sensitivity results can be summarized as follows:

(1) The ocean-going vessels sector is the optimal alternative for all possible scenarios;
(2) In comparing the refineries sector and the electricity-generating units sector, when the scenarios fall in the area of y < 0.5 (implementation difficulty’s weight smaller than 0.5), refineries are more preferred;
(3) A decision-maker’s attitude towards each criterion is closely related to the decision-making process and can certainly influence their final decision.

## 8. Conclusions and Policy Implications

We investigated how a science-based decision can be made in terms of mitigating future PM_2.5_ increase risk in LA-LBMA using a risk and decision analysis framework. Generally, we first chose economic growth risk as to the section that we wanted to make our decision on, based on a risk analysis process. Quantitative decision analysis suggested that refineries should be preferred for investment, while a multi-criterion decision analysis process suggested that the ocean-going vessels are the worthiest alternative to invest in.

The risk analysis in this paper took population growth, economic growth, and climate change into consideration. Results of the risk analysis revealed that economic growth is the most important decision factor in this case. Improvement can be made on incorporating more accurate data associated with an exact probability of risk factors, more updated simulation model results, and more accurate predictions of the range of PM_2.5_ concentration increase in the future.

The decision analysis in this paper focused on three sectors out of 17 sectors to choose the prior sector to invest under the circumstance of limited resources. Quantitative and qualitative analyses were carried out based on different criteria. Both EMV and EU methods found that the refineries sector should be the priority for investment. However, when we incorporated three potential criteria, the optimal alternative changed to the ocean-going vessels sector. Sensitivity analysis indicated that decision-makers’ attitude towards different criterion are of great influence on their final decision-making process. Further analysis can take place in incorporating more potential criteria in the decision-making process, and also in terms of analyzing more alternatives to get a comprehensive picture of the decision-making problem.

Specifically, for LA-LBMA, we retain optimistic attitude towards the region’s future air quality. Efforts can be made in several aspects:

(1) Control the population in the region—it is reasonable to think that fewer people will reduce the PM_2.5_ emission activities. The risk analysis in our case study shows that population expansion remains the second severe risk factor in our study scope. However, for the reason that a close connection exists between the population expansion and economic growth, we believe controlling population expansion in the LA-LBMA region is an efficient approach to alleviate future air pollution stress on the region;
(2) Partner with other local governments to deal with the climate change issue—climate change is a broader challenge faced by countries all over the world. Thus, cooperation with others becomes extremely important. Our case study only considers the temperature increase as the only element of climate change. For a coastal region like LA-LBMA, we believe that custom policies that help adapt to climate change are necessary for controlling the air quality risk;
(3) Increase the investment efficiency—efforts can be made in policies to help the funds associated with controlling PM_2.5_ emissions be more efficient and effective. The sensitivity analysis in our case study showed that the decision criterion plays a critical role in making the final decision. The subjective-related criterion such as investment efficiency that can be controlled by local officials is vital to help advance air pollution mitigation efforts;
(4) Increase the investment in technology development—technology is the key to refining sectors to reduce emissions. For example, the agencies associated with two ports in the Long Beach area have issued programs to support the development of emission constraint technologies and have already obtained great success. Besides, based on the decision analysis in our case study, the ocean-going vessels sector is the optimal alternative to invest in and transform, and we are confident that a continuous increase in investment in refining the energy use efficiency of these ocean-going vessels will bring benefits to LA-LBMA’s air quality in the long term;
(5) Think about other potential risk factors that need attention—this study only considers three potential factors: population, economy, and climate. There are certainly other factors that can directly or indirectly influence the PM_2.5_ emissions. Finding them and incorporating them into the current risk and decision analysis framework should be the future research direction.

This study proposes a practicable risk and decision analysis framework that can fast ensemble experts’ knowledge and opinions as well as simulation data from other studies to help local officials inform science-based decisions associated with alleviating future air pollution stress. We acknowledge that the case study presented here simplifies the real-world decision problem context and only considers very limited risk factors and alternatives in each risk factor. As a result, the results from our case study might not be sufficient to directly support real-world scenarios as the decision-making process is closely dependent on different decision problem contexts faced by decision-makers. Moreover, the efficiency and feasibility of this proposed risk and analysis framework have not been tested when more risk factors and more alternatives are present, as well as when more complicated decision criteria are incorporated. Future research should be focused on upgrading this proposed risk and decision analysis framework to adapt to more complex decision problems and contexts in terms of the model’s efficiency and reliability.

## Figures and Tables

**Table 1:** Summary of Air Quality Programs.

Program	Characteristics
Clean Air Action Plan (CAAP)	Strategies to reduce health risks to communities
San Pedro Bay Standards	The updated CAAP with the addition of the SPBS, which represents the health risk and emissions reduction goals
Technology Advancement Program	Identify and demonstrate new emissions reduction technologies
Clean Trucks Program	Reduced air pollution from harbor trucks by more than 90%
Green Flag Program	Voluntary vessel speed reduction
Green Ship Incentive Program	Bring the newest, cleanest ships to the port
Shore Power	Require ships to plug into the electrical grid
Clean Air Action Plan Awards	A strong commitment to reducing air pollutant emissions from port-related sources
Annual Air Emissions Inventories	Conducts an inventory of air emissions from port-related sources
Air Quality Monitoring	Operates an air quality monitoring network that collects data on ambient air quality

Source: Port of Long Beach [36].”Reproduced with permission from Port of LONG BEACH, Air Quality Programs Details, published by Port of LONG BEACH, 2020.”

**Table 2:** Defined increase level based on average annual increase rate of impact factors.

Factors	Increase Rate *	Definition of Increase level *
Population	0.6–1%	High
	0.3–0.6%	Medium
	0–0.3%	Low
Temperature	1–1.5%	High
	0.5–1%	Medium
	0–0.5%	Low
Economic	1–2%	High
	0.5–1%	Medium
	0–0.5%	Low

* We consulted experts’ opinions regarding the increase rate and the definition of the increase level for each risk factor.

**Table 3:** Defined level of PM_2.5_ concentration increase within 10 years.

Increase Level	Increase Rate	PM_2.5_ Concentration
High	20%	14.4 µg/m^3^
Medium	10%	13.2 µg/m^3^
Low	5%	12.6 µg/m^3^

**Table 4:** Conditional probability associated with risk rating.

P[Consequence = High | Risk Factor = (High, Medium, Low)]	P(Risk Factor = High | Consequence = High)
P(consequence = high | population growth = high) = 0.41	P(population growth = high | consequence = high) = 0.4184
P(consequence = high | population growth = medium) = 0.0756	
P(consequence = high | population growth = low) = 0	
P(consequence = high | economic growth = high) = 0.4256	P(economic growth = high | consequence = high) = 0.608
P(consequence = high | economic growth = medium) = 0.06	
P(consequence = high | economic growth = low) = 0	
P(consequence = high | temperature increase = high) = 0.23616	P(temperature increase = high | consequence = high) = 0.1976
P(consequence = high | temperature increase = medium) = 0.10944	
P(consequence = high | temperature increase = low) = 0.14	

**Table 5:** Monetary value per ton of directly emitted PM_2.5_ reduced from each sector.

Sector	Monetary Value of Directly Emitted PM_2.5_ Reduced from Sectors $/ton
Ocean-going vessels	$53,000
Refineries	$370,000
Electricity-generating units	$150,000

**Table 6:** Potential consequence of PM_2.5_ concentration increase.

Projection of PM_2.5_ Increase	Probability	PM_2.5_ Concentration Needed to be Reduced	Mass of PM_2.5_ Needed to Be Reduced
High	0.49	2.4 µg/m^3^	2.4 × 10^−12^ V ton
Medium	0.42	1.2 µg/m^3^	1.2 × 10^−12^ V ton
Low	0.097	0.6 µg/m^3^	0.6 × 10^−12^ V ton

**Table 7:** Probabilities of PM_2.5_ increase and success of each scenario for each alternative.

Sector	Magnitude of PM_2.5_ Increase	Probabilities of Magnitude of PM_2.5_ Increase for Each Sector *	Probabilities of Success *
Ocean-going vessels	High	0.4	0.95
	Medium	0.38	0.9
	Low	0.22	0.9
Refineries	High	0.5	0.75
	Medium	0.4	0.8
	Low	0.1	0.5
Electricity-generating units	High	0.55	0.6
	Medium	0.4	0.5
	Low	0.05	0.3

* Numbers are derived from experts’ opinions and assumptions.

**Table 8:** Summary of EMV calculation results.

Scenario	Probability	Mass of PM_2.5_ Needed to Be Reduced	Monetary Value for Each Scenario (Probability of Success Times Monetary Value)	EMV
**Ocean-Going Vessels**				
High	0.4	2.4 × 10^−12^ V	1.21 × 10^−7^ V	7.64 × 10^−8^ V
Medium	0.38	1.2 × 10^−12^ V	5.72 × 10^−8^ V	
Low	0.22	0.6 × 10^−12^ V	2.86 × 10^−8^ V	
**Refineries**				
High	0.5	2.4 × 10^−12^ V	6.66 × 10^−7^ V	4.86 × 10^−7^ V
Medium	0.4	1.2 × 10^−12^ V	3.55 × 10^−7^ V	
Low	0.1	0.6 × 10^−12^ V	1.11 × 10^−7^ V	
**Electricity-Generating Units**				
High	0.45	2.4 × 10^−12^ V	2.16 × 10^−7^ V	1.37 × 10^−7^ V
Medium	0.4	1.2 × 10^−12^ V	0.9 × 10^−7^ V	
Low	0.15	0.6 × 10^−12^V	2.7 × 10^−8^V	

**Table 9:** Calculation of EU with various risk tolerance (R) value.

R1 = 5 (Risk Averse)			R2 = 100 (Risk Neutral)			R3 = 200 (Risk Tolerance)		
Ocean-Going Vessels	Utility	EU	Ocean-Going Vessels	Utility	EU	Ocean-Going Vessels	Utility	EU
High	2.41 × 10^−8^	1.52 × 10^−8^	High	1.21 × 10^−9^	4.84 × 10^−10^	High	0	0
Medium	1.14 × 10^−8^		Medium	0		Medium	0	
Low	5.72 × 10^−9^		Low	0		Low	0	
**Refineries**			**Refineries**			**Refineries**		
High	1.33 × 10^−7^	9.72 × 10^−8^	High	6.66 × 10^−9^	4.86 × 10^−9^	High	3.33 × 10^−9^	2.43 × 10^−9^
Medium	7.10 × 10^−8^		Medium	3.55 × 10^−9^		Medium	1.78 × 10^−9^	
Low	2.22 × 10^−8^		Low	1.11 × 10^−9^		Low	5.6 × 10^−10^	
**Electricity-generating units**			**Electricity-generating units**			**Electricity-generating units**		
High	4.32 × 10^−8^	2.75 × 10^−8^	High	2.16 × 10^−9^	1.37 × 10^−9^	High	1.08 × 10^−9^	6.66 × 10^−10^
Medium	1.8 × 10^−8^		Medium	0.9 × 10^−9^		Medium	4.5 × 10^−10^	
Low	5.4 × 10^−9^		Low	2.7 × 10^−10^		Low	0	

**Table 10:** Weight of each criterion.

Criteria	Weight *
Investment efficiency	0.6
Implementation difficulty	0.3
Time to become effective	0.1

* The weight assigned in this study is an example and they can certainly be changed by the decision-maker based on their criterion rating.

**Table 11:** Ranges of valuation for criteria and ranking.

**Investment Efficiency ($ M/ton)**	**Rank**
100–300	1
300–500	2
500–700	3
700–900	4
**Implementation Difficulty**	**Rank**
Easy	1
Medium	2
Hard	3
Extremely hard	4
Time to Become Effective (Years) *	**Rank**
0–5	1
5–10	2
10–15	3
15–20	4

* The time to become effective are estimated based on experts’ opinions and assumptions.

**Table 12:** Alternative preference for multi-criteria decision analysis.

Sector\Criteria	Investment Efficiency ($ M/ton)	Implementation Difficulty	Time to Become Effective (Years)	Score
Ocean-going vessels	100–300 (score = 4)	Medium (score = 3)	0–5 (score = 4)	3.7
Refineries	500–700 (score = 2)	Hard (score = 2)	5–10 (score = 3)	2.1
Electricity-generating units	700–900 (score = 1)	Medium (score = 3)	10–15 (score = 2)	1.7

**Table 13:** Five different scenarios of weight assignment.

Criteria	Weight (Scenario 1)	Weight (Scenario 2)	Weight (Scenario 3)	Weight (Scenario 4)	Weight (Scenario 5)
Investment	0.8	0.7	0.6	0.5	0.3
efficiency					
Implementation difficulty	0.1	0.2	0.2	0.3	0.4
Time to become effective	0.1	0.1	0.2	0.2	0.3

## Data Availability

The data presented in this study are available on request from the corresponding author. The data are not publicly available due to the inclusion of information that compromises the privacy of research participants.

## Supplementary Materials

The following are available online at https://www.mdpi.com/article/10.3390/ijerph18094905/s1, supplementary file.
