# Supplementary material for: A Risk and Decision Analysis Framework to Evaluate Future PM2.5 Risk: A Case Study in Los Angeles-Long Beach Metro Area
Source: Int J Environ Res Public Health. 2021 May 4;18(9):4905. doi: 10.3390/ijerph18094905 (PMC8124696; doi:10.3390/ijerph18094905)
Supplement: Supplementary file 1 [file ijerph-18-04905-s001.zip › ijerph-1180432-supplementary.pdf]

Supporting Information for

A Risk and Decision Analysis Framework to Evaluate Future $PM_{2.5}$ Risk: A Case Study in Los Angeles-Long Beach Metro Area

Bowen He [1,*] and Qun Guan [2]

[1] Department of Civil and Environmental Engineering, Vanderbilt University, Nashville, Tennessee, 37235, USA.
[2] College of Civil Engineering, Hefei University of Technology, Hefei, Anhui, 230009, China

Introduction

This study proposes a valuable risk and decision analysis framework for analyzing the air pollution problem associated with the future $PM_{2.5}$ concentration increase caused by three risk factors: population growth, economic growth, and climate change. Here we provide additional information regarding the expert's opinion consultation process. In this study, we formed a committee of experts consists of a total of 20 people who specialize in the demographic census, economic analysis and prediction, and climate change field, respectively. Table S1 summarizes the information regarding the experts' committee in this study. For the demographic census area of expertise, we consulted 8 experts with 6 professors and 2 government officials. For economic analysis, we consulted 7 experts with 5 professors and 2 private sector persons. In terms of climate change, we consulted 5 experts with 5 professors from a private research university.

Table S1. Summary of experts committee.

Area of Expertise	Number of Experts	Profession
Demographic Census	8	Professor (6), Government official (2)
Economic Analysis	7	Professor (5), Private sector persons (2)
Climate Change	5	Professor (5)

In order to obtain the approximated cumulative probabilities for three risk factors associated with future $PM_{2.5}$ concentration increase in LA-LBMA, we create a questionnaire separately for each risk factor. Then, we collect answers for these questionnaires from corresponding experts of that specific area of expertise. Then, the answers are averaged from those experts from each area of expertise to generate an approximated cumulative probability function for each risk factor (Figure 7 and Table 2). The questionnaire for experts for each risk factor is summarized in Table S2.

Table S2. Summary of questionnaire regarding the cumulative probability of each risk factor.

Risk Factor	Question
Population Expansion	(1) In your opinion, what is the largest possible population increase rate in the LA-LBMA within the future 10 years?
	(2) In your opinion, what is the probability that the population increase rate in the LA-LBMA will be less than the 75th percentile of the largest possible population increase rate based on your answer to the question (1)?
	(3) In your opinion, what is the probability that the population increase rate in the LA-LBMA will be less than the 50th percentile of the largest possible population increase rate based on your answer to the question (1)?
	(4) In your opinion, what is the probability that the population increase rate in the LA-LBMA will be less than the 25th percentile of the largest possible population increase rate based on your answer to the question (1)?

	(5) In your opinion, what is the range of the population increase rate that you would consider as a high increase scenario?
	(6) In your opinion, what is the range of the population increase rate that you would consider as a medium increase scenario?
	(7) In your opinion, what is the range of the population increase rate that you would consider as a low increase scenario?
Temperature Increase	(1) In your opinion, what is the largest possible temperature increase rate in the LA-LBMA within the future 10 years?
	(2) In your opinion, what is the probability that the temperature increase rate in the LA-LBMA will be less than the 75th percentile of the largest possible temperature increase rate based on your answer to the question (1)?
	(3) In your opinion, what is the probability that the temperature increase rate in the LA-LBMA will be less than the 50th percentile of the largest possible temperature increase rate based on your answer to the question (1)?
	(4) In your opinion, what is the probability that the temperature increase rate in the LA-LBMA will be less than the 25th percentile of the largest possible temperature increase rate based on your answer to the question (1)?
	(5) In your opinion, what is the range of the temperature increase rate that you would consider as a high increase scenario?
	(6) In your opinion, what is the range of the temperature increase rate that you would consider as a medium increase scenario?
	(7) In your opinion, what is the range of the population increase rate that you would consider as a low increase scenario?
GDP Increase	(1) In your opinion, what is the largest possible GDP increase rate in the LA-LBMA within the future 10 years?
	(2) In your opinion, what is the probability that the GDP increase rate in the LA-LBMA will be less than the 75th percentile of the largest possible temperature increase rate based on your answer to the question (1)?
	(3) In your opinion, what is the probability that the GDP increase rate in the LA-LBMA will be less than the 50th percentile of the largest possible temperature increase rate based on your answer to the question (1)?
	(4) In your opinion, what is the probability that the GDP increase rate in the LA-LBMA will be less than the 25th percentile of the largest possible temperature increase rate based on your answer to the question (1)?
	(5) In your opinion, what is the range of the GDP increase rate that you would consider as a high increase scenario?
	(6) In your opinion, what is the range of the GDP increase rate that you would consider as a medium increase scenario?
	(7) In your opinion, what is the range of the GDP increase rate that you would consider as a low increase scenario?

In the quantitative decision analysis section, we also consulted experts from the economic analysis and the climate change field to estimate the probabilities of success to refine each alternative. The answers are averaged to indicate an approximated number. The detailed questionnaire is summarized in Table S3.

Table S3. Summary of questionnaire regarding the probabilities of success for each alternative and the approximated time needed to become effective.

Alternative	Questionnaire
Ocean-going vessels	(1) In your opinion, what is the probability of success to transform the ocean-going vessels sector in LA-LBMA when the $PM_{2.5}$ concentration undergoes a high increase scenario?
	(2) In your opinion, what is the probability of success to transform the ocean-going vessels sector in LA-LBMA when the $PM_{2.5}$ concentration undergoes a high increase scenario?
	(3) In your opinion, what is the probability of success to transform the ocean-going vessels sector in LA-LBMA when the $PM_{2.5}$ concentration undergoes a medium increase scenario?
	(4) In your opinion, how long will the ocean-going vessels need to be transformed to reduce the $PM_{2.5}$ emissions in the LA-LBMA?
Refineries	(1) In your opinion, what is the probability of success to transform the refineries sector in LA-LBMA when the $PM_{2.5}$ concentration undergoes a high increase scenario?
	(2) In your opinion, what is the probability of success to transform the refineries sector in LA-LBMA when the $PM_{2.5}$ concentration undergoes a medium increase scenario?
	(3) In your opinion, what is the probability of success to transform the refineries sector in LA-LBMA when the $PM_{2.5}$ concentration undergoes a low increase scenario?
	(4) In your opinion, how long will the refineries need to be transformed to reduce the $PM_{2.5}$ emissions in the LA-LBMA?
Electricity-generating units	(1) In your opinion, what is the probability of success to transform the electricity-generating units sector in LA-LBMA when the $PM_{2.5}$ concentration undergoes a high increase scenario?
	(2) In your opinion, what is the probability of success to transform the electricity-generating units sector in LA-LBMA when the $PM_{2.5}$ concentration undergoes a medium increase scenario?
	(3) In your opinion, what is the probability of success to transform the electricity-generating units sector in LA-LBMA when the $PM_{2.5}$ concentration undergoes a low increase scenario?
	(4) In your opinion, how long will the electricity-generating units need to be transformed to reduce the $PM_{2.5}$ emissions in the LA-LBMA?
